# Combined Lacrimal Passage Probing and Tobramycin/Dexamethasone Ophthalmic Ointment Infiltration: A Minimally Invasive Surgical Procedure for Incomplete Nasolacrimal Duct Obstruction

**DOI:** 10.1097/MD.0000000000001483

**Published:** 2015-09-11

**Authors:** Jianjiang Xu, Jiaxu Hong, Xinghuai Sun, Zuguo Liu, Alireza Mashaghi, Takenori Inomata, Yi Lu, Yimin Li, Dan Wu, Yujing Yang, Anji Wei, Yujin Zhao, Chun Lu

**Affiliations:** From the Department of Ophthalmology and Visual Science (JX, JH, XS, Yi Lu, Yimin Li, DW, YY, AW, YZ, CL), Eye, Ear, Nose and Throat Hospital, Shanghai Medical College, Fudan University, Shanghai, China; School of Life Sciences (JH, ZL), Xiamen University, Fujian Provincial Key Laboratory of Ophthalmology and Visual Science, Fujian, China; Massachusetts Eye and Ear Infirmary (JH, AM), Harvard Medical School, Boston, Massachusetts; China State Key Laboratory of Medical Neurobiology (XS), Institutes of Brain Science, Shanghai, China; Key Laboratory of Myopia (XS), National Health and Family Planning Commission, Shanghai, China; and Department of Ophthalmology (TI), Juntendo University School of Medicine, Tokyo, Japan.

## Abstract

The optimal treatment strategy for an incomplete nasolacrimal duct obstruction (INDO) is still being debated. The aim of this study is to evaluate the treatment results of combined lacrimal passage probing and tobramycin/dexamethasone ophthalmic ointment infiltration (PIO, Probing and Injection) for INDO.

In this retrospective, noncomparative case series, 397 consecutive adult patients with INDO treated at Shanghai Eye, Ear, Nose and Throat Hospital were enrolled. Records of the patients were reviewed. With the help of a modified 23-gauge lacrimal cannula, the PIO surgery was performed for the INDO-identified patients. The main outcome measures were resolution of tearing and complications. The relationship between successful outcome and clinical characteristics was analyzed.

The surgery was performed successfully in all of the enrolled cases. No intraoperative complications were found in the procedure. The average follow-up time was 7.9 months. Three hundred patients (75.6%) experienced complete resolution of their symptoms after the surgery. Ninety-seven patients (24.4%) showed a partial improvement (1.8%), no improvement (18.4%), or a worsening of symptoms (4.3%). Of the 97 surgical-failure patients, 90 required silicone intubation or external dacryocystorhinostomy, and 94% were finally resolved. The most common postoperative complications were mild nasal bleeding in 41 patients, drug residues in 12 patients (6 developed the complete obstruction), and a slit punctum in 8 patients. Multivariate logistic regression analysis revealed that unilateral eye onset, not having a discharge at baseline, and not having postoperative drug residues were significant factors determining successful outcome.

The PIO surgery is an effective, safe, timesaving, easy-to-perform, and minimally invasive technique for treating INDO.

## Abstract 2

Supplemental Digital Content is available in the text

## INTRODUCTION

Nasolacrimal duct obstruction causes a watering of the eyes, and leads to patient discomfort and decreased visual-related quality of life.^1,2^ Dacryocystorhinostomy (DCR) is a common procedure for primary acquired nasolacrimal duct obstruction, and can be performed through a cutaneous incision (external DCR), or via a transnasal approach under either direct visualization or endoscopic guidance (endonasal DCR). Although DCR surgery is currently the best procedure for a complete nasolacrimal duct obstruction with a ≥90% success rate,^3^ the optimal treatment strategy for an incomplete nasolacrimal duct obstruction (INDO) is still being debated.

Surgical management strategies for INDO include external or endonasal DCR, balloon catheter dilatation, and silicone intubation. Although the reported success rate of DCR for INDO has been as high as 85%,^4^ few surgeons and patients consider it as the first choice because of the associated nasal bleeding, incisional scarring, and other complications. In practice, less-invasive interventions, including balloon catheter dilatation and silicone intubation, are recommended for treating INDO, though they have lower overall success rates, ranging from 52% to 85% (Supplemental file 1, http://links.lww.com/MD/A419).^5–8^ These estimates were made nearly a decade ago in a series of published studies that include relatively a small number of patients. In this article, we introduce the combined lacrimal passage probing and tobramycin/dexamethasone ophthalmic ointment infiltration (PIO, Probing and Infiltration) with a modified lacrimal cannula set as a novel method to treat INDO, and report on the results of a large case series composed of 397 patients who received this therapy.

## METHODS

All of the patients who were diagnosed with INDO and underwent the PIO surgery at Shanghai Eye, Ear, Nose and Throat Hospital between May 2011 and May 2014 were included in this study. The study was approved by the Institutional Review Board and the Medical Ethics Committee of the hospital. Written informed consent was obtained from all of the participants before the surgery, and the project was conducted in adherence with the tenets of the Declaration of Helsinki. The medical records were reviewed retrospectively, and the patient demographics, symptoms and signs, intraoperative findings, and clinical outcomes were collected. The INDO patients included in this study were those who showed symptoms of tearing or discharge/mattering accompanied by dye-disappearance testing (negative Jones I test results and positive Jones II test results), nasolacrimal system irrigation (fluid reflux through the opposite canaliculus and late and little passage into the nose), and the absence of other ocular surface diseases known to induce epiphora.^6,7^ The dye-disappearance test was performed with instillation of a drop of 2% fluorescein sodium placed in the conjunctival fornix and assessed after 5 minutes by putting a cotton bud soaked in anesthetic in the inferior meatus to see whether fluorescein is detected. If no fluorescein is discovered, this is a negative Jones I test. Next, we washed the excess fluorescein from the conjunctival sac and syringe. If fluorescein is detected, then this is a positive Jones II test and suggests a functional obstruction of the nasolacrimal duct. Those patients who had previous nasolacrimal surgery or were diagnosed with complete nasolacrimal duct obstruction were excluded from this study.

**FIGURE 1:**
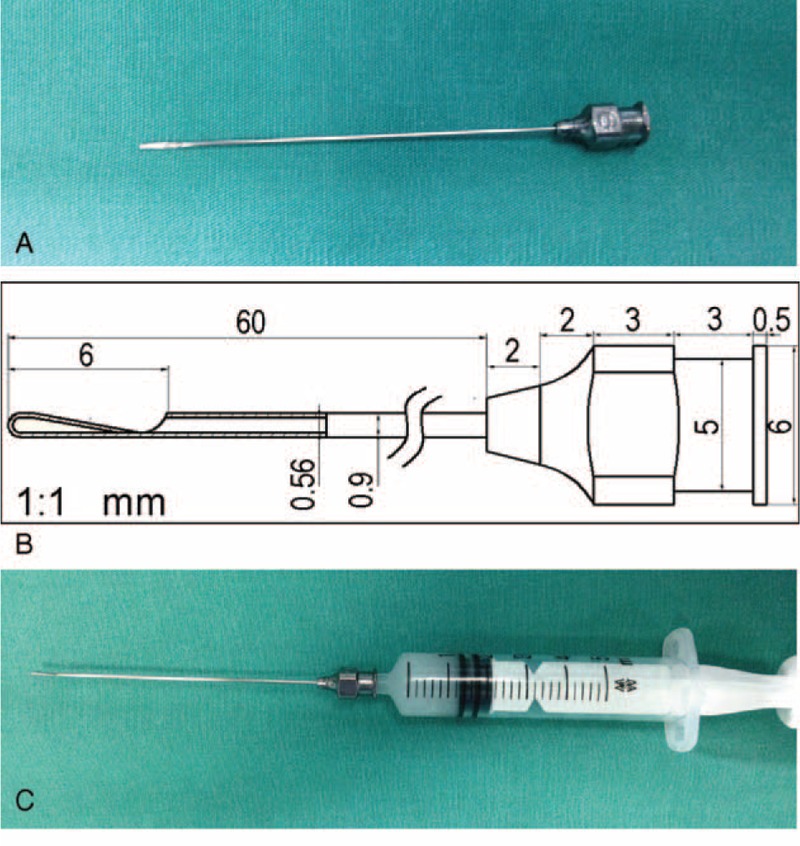
Instruments are shown for combined lacrimal passage probing and tobramycin/dexamethasone ophthalmic ointment infiltration. (A) A modified 23-gauge stainless steel lacrimal cannula is 70.5 mm in length with a blunt conical tip and an injection orifice on the front side; the cannula was well polished. (B) This modified lacrimal cannula, with a 0.56 mm inner diameter, 0.9 mm outer diameter, and 70.5 mm length, has a smooth and blunt blind tip with a 6 mm lateral opening above the tip. (C) The modified 23-gauge lacrimal cannula is connected to a 5 mL injector with 1 to 1.5 mL tobramycin 0.3% and dexamethasone 0.1% ophthalmic ointment, as is shown.

The surgical procedure involved the following steps (see Supplemental files 2 to 4, http://links.lww.com/MD/A420, http://links.lww.com/MD/A421, http://links.lww.com/MD/A422 for surgical videos of 3 different patients (**Supplemental Videos 1-3** Surgical videos of three different incomplete nasolacrimal duct obstruction patients. The surgical procedure was shown in the text of the videos. Briefly, the surgery was performed in the office-based and operating room settings. Patients were required to lie face up on their back. At the beginning of the surgery, 1 mL of lidocaine 2% without epinephrine was injected into the nasolacrimal duct for the infiltration anesthesia. Then, both the upper and lower punctum were dilated. A modified 23-gauge lacrimal cannula set was used in this procedure. The stainless steel lacrimal cannula is 70.5 mm long with a blunt, conical tip and an injection orifice on the front side; the cannula is well polished. The canaliculi and nasolacrimal duct were probed with the modified lacrimal cannula via the upper punctum. The tip of the cannula passed through the nasolacrimal duct and entered into the inferior meatus and had a hard stop. The tobramycin 0.3% and dexamethasone 0.1% ophthalmic ointment (1∼1.5 mL) was injected while the tips went backward. After the ointment flowed out of the lower punctum, the surgery was complete.. The surgery was performed in the office-based and operating room settings. Patients were required to lie face up on their back. At the beginning of the surgery, 1 mL of lidocaine 2% without epinephrine was injected into the nasolacrimal duct for the infiltration anesthesia. Then, both the upper and lower punctum were dilated. A modified 23-gauge lacrimal cannula set was used in this procedure (see Figure 1). The stainless steel lacrimal cannula is 70.5 mm long with a blunt, conical tip and an injection orifice on the front side; the cannula is well polished. The canaliculi and nasolacrimal duct were probed with the modified lacrimal cannula via the upper punctum. The tip of the cannula passed through the nasolacrimal duct and entered into the inferior meatus and had a hard stop. The tobramycin 0.3% and dexamethasone 0.1% ophthalmic ointment (1–1.5 mL, Tobradex ®, Alcon, Fort Worth, USA) was injected while the tips went backward. After the ointment flowed out of the lower punctum, the surgery was complete. Postoperatively, the patients received topical 0.3% tobramycin/0.1% dexamethasone eye drops 3 times per day for 2 weeks, and topical 0.3% tobramycin eye drops (Tobradex, Alcon Laboratories) 3 times per day for another 2 weeks.

The patients required follow-up appointments for at least 6 months. The first lacrimal irrigation was routinely administered to clear up the potential drug residues in the lacrimal passage in the outpatient department at 1 month after surgery. The patients were then monitored during follow-up appointments. The clinical outcomes were categorized as follows:^6,7^ (1) success—the disappearance of the symptoms and signs; (2) failure—a partial improvement (occasional epiphora and some reflux on lacrimal irrigation), no improvement, or a worsening of symptoms (no significant improvement in epiphora postoperatively, and had significant reflux during irrigation).

An analysis was performed using the statistical software package SPSS for Windows (version 19.0; SPSS Inc, Chicago, IL). In cases with bilateral surgeries, only the first eye was enrolled for analysis. The normal distribution data are shown as mean ± standard deviation. For comparative purposes, the mean values differences of the groups’ parametric data were analyzed using the Student *t* test or the Mann–Whitney *U* test. The percentage of parameters between different groups was analyzed with a χ^2^ test. All of the variables were entered into a binary logistic regression analysis one at a time; variables with a probability of 0.2 or less for a relationship with the outcome of the complete resolution were entered into a multivariate logistic regression analysis. All of the *P* values were 2-sided and considered statistically significant when the values were <0.05.

## RESULTS

During the study, 857 patient medical records reported a nasolacrimal duct obstruction. Of these, 394 did not meet the inclusion criteria (351 had a complete obstruction and 43 had a previous lacrimal duct surgery), and 24 had the follow-up of <6 months. Forty-two patients chose a topical medication treatment rather than the surgery. Overall, 397 patients were suitable for the study.

As shown in Table 1, the PIO surgery was successfully performed in 397 eyes of 397 patients (1 eye per patient) whose ages averaged 58.5 ± 14.3 years (a range of 17–93). There were 98 men (24.7%) and 299 women (75.3%). Overall, 242 patients (61%) had unilateral INDO and 155 (39%) had bilateral INDO.

**TABLE 1:**
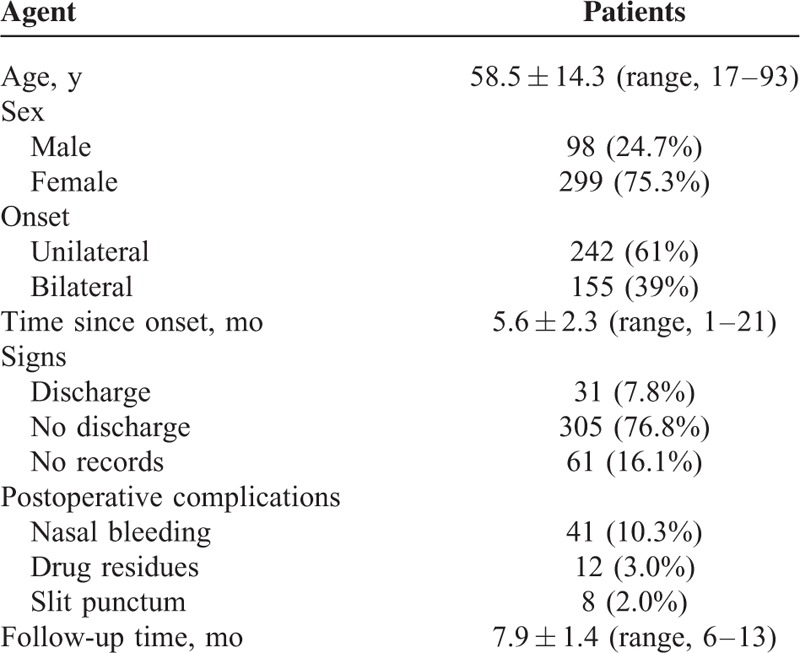
Demographic and Clinical Data of Patients With Incomplete Nasolacrimal Duct Obstruction (n = 397)

The tearing symptom presented in all of the eyes. The second prominent symptom/sign was discharge, including spontaneous discharge and discharge induced by massage, which was present in 31 eyes (7.8%). The average follow-up time was 7.9 ± 1.4 months (a range of 6–13). Postoperatively, nasal bleeding was reported in 41 patients during follow-up visits. Drug residues were identified in 12 patients, 6 of whom developed a complete nasolacrimal obstruction. A slit punctum was observed in 8 eyes. No other complications were seen.

An overall success rate of 75.6% (300/397) was achieved during the final follow-up visit, as shown in Table 2. In addition, only 7 patients (7/397, 1.8%) were partially improved after surgery. Seventy-three patients (73/397, 18.4%) had no improvement, and 17 patients (17/397, 4.3%) even became worse. There was no statistically significant difference in the age, sex, and follow-up time between the success and the failure groups. However, patients in the success group seemed to have more unilateral involvement (*P* = 0.008), a shorter duration of onset (*P* = 0.03), less discharge at the baseline (*P* = 0.001), and fewer postoperative complications (*P* < 0.001). Among the 97 surgical-failure patients, 90 required more surgical intervention and 94% were finally resolved. The patients with postoperative drug residues were only found in the failure group.

**TABLE 2:**
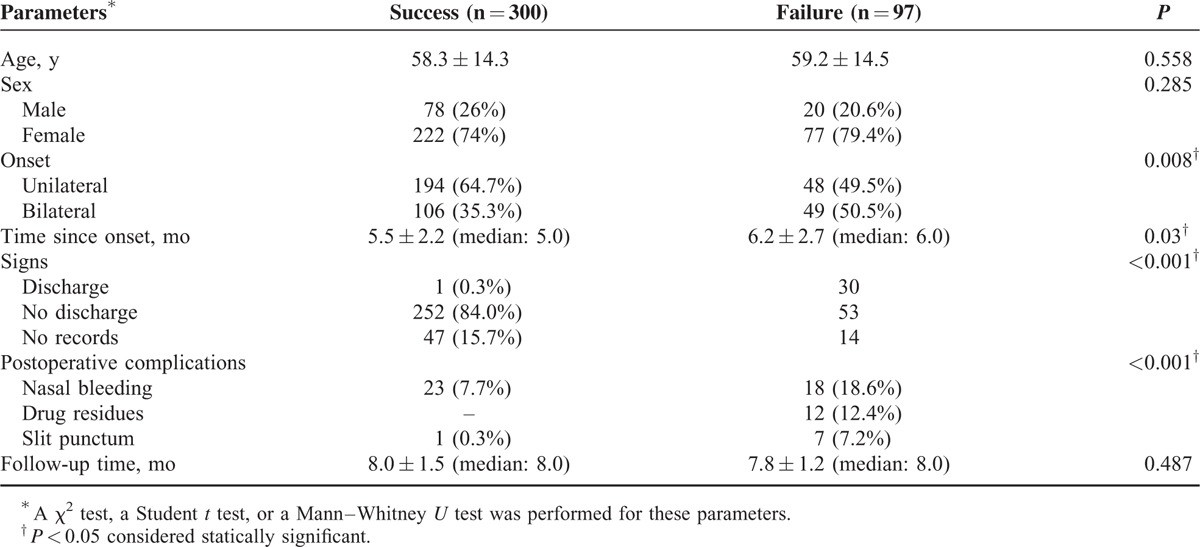
Clinical Characteristics of Patients With Different Prognoses

Multivariate linear regression model revealed the following factors (see Table 3) to have a statistically significant positive effect on the success rates: unilateral eye onset (*P* = 0.011), not having a discharge at baseline (*P* < 0.001), and not having postoperative complications (*P* = 0.001), especially drug residues (*P* = 0.04). Age, sex, time since onset, the occurrence of postoperative nasal bleeding or slit punctum, and a longer follow-up time were not significant factors.

**TABLE 3:**
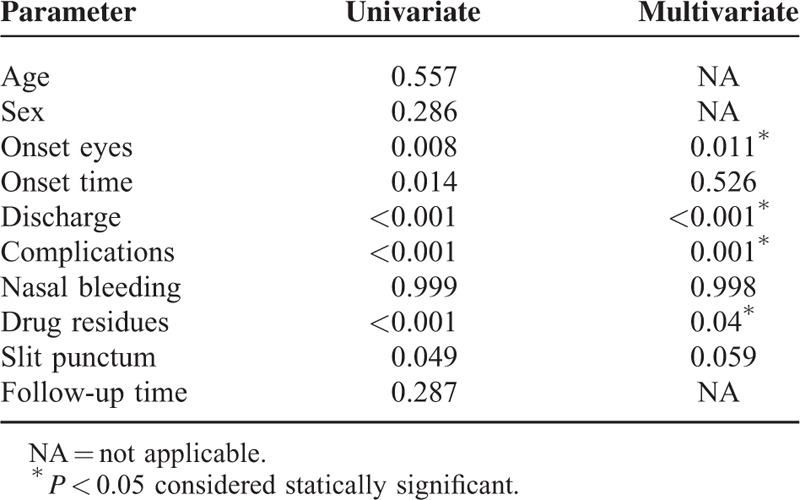
Univariate and Multivariate Regression Analysis on Potential Factors That Influence Surgical Success

## DISCUSSION

Our modified surgical set provides a technique for performing the lacrimal passage probing and the infiltration of tobramycin/dexamethasone ophthalmic ointment at one time, which simplifies the operation procedure, reduces the surgical time, and minimizes the injury caused by the surgery. The success rate in our study was comparable to that of other reports,^5–10^ and the incidence of postoperative complications was acceptable.

The most common initial treatment for INDO is silicone intubation.^11^ Unfortunately, its success rate is relatively low, compared with the DCR. With single silicone intubation, Keith and Dortzbach reported 73% to 74% success rates in small case series of INDO patients.^9,10^ The other studies showed that single silicone intubation, with or without a balloon dacryocystoplasty, resolved the symptoms in 47% to 61% of patients during a long-term follow-up.^6,12,13^ Using a “double silicone tube intubation” technique, Demirci and Elner achieved a relatively high success rate of 79% in 24 eyes of the 18 consecutive adult patients.^14^

Based on our case series, which is the largest to this date, the PIO surgery compares favorably with the reported success rates of the single silicone intubation treatment. More importantly, this method could be performed in an office setting. A direct comparison between the results of the previous studies and our study may be impossible because of the variability in patient race, sample size, follow-up periods, and surgical techniques. One possible reason for the favorable success rate may be that the ointment includes antibiotics and immunosuppressive agents, which are effective for directly controlling the local inflammation. A recent human anatomy study reported the conspicuous inflammation and exudate in the narrowed portions of the nasolacrimal duct.^15^ The ointment used in the surgery may have helped ameliorate the inflammation and reduce the risk of recurrence. In addition, the dexamethasone could inhibit the proliferation of fibroblast during the wound repair, preventing the restenosis of the nasolacrimal duct.^16^ Further prospective and controlled studies are warranted to demonstrate consistent outcomes using our surgical method.

Despite the minimum damage to the lacrimal passage, a few mild and mostly self-limited complications were found after the PIO surgery. These complications included nasal bleeding (10.3%), drug residues (3%), and slit punctum (2%). None of 41 nasal bleeding patients in the present series required surgical interventions. The 12 patients who received postoperative lacrimal syringing were found to have drug residues; 6 of them showed no improvement, and the other 6 patients worsened and developed complete nasolacrimal duct obstruction. This begs the question of why the drug residues occurred in some of patients. The exact kinetics of the ointments in the nasolacrimal duct is unknown, yet most of the patients who received lacrimal syringing 1 month after the surgery reported no drug residues. It has been reported that lipogranuloma can develop in patients who received transcanalicular ointment injection after laser canaliculoplasty.^17^ In those patients, the ointment was injected to a new artificial channel which was created by laser canaliculoplasty, meaning it was hard to be removed. This potential safety issue needs to be addressed in the future, although it did not happen to our patients. Eight eyes with slit punctum may have resulted from vigorous dilation, which has limited effect on the symptoms of the patients because the opposite punctum was usually normal.

We also identified the following significant factors associated with surgical success by multivariate analysis: unilateral eye onset, not having a discharge at baseline, and not having postoperative drug residues. Few articles explored the risk factors of the failure in the surgical procedures for INDO. Previously, bilateral congenital nasolacrimal duct obstruction was considered to be a poor prognostic factor because it could indicate a significant anatomical variation in the lacrimal duct that cannot be successfully resolved with probing.^18^ Having a discharge before the PIO surgery reminded us that there was an infection of unknown cause in these patients. It has been reported that the patients’ history of infectious conjunctivitis was one of the predisposing factors for the primary acquired nasolacrimal duct obstruction.^19^ Patients with postoperative drug residues may have relatively narrow nasolacrimal ducts. For them, DCR or silicone intubation may be more suitable than the PIO surgery. Other factors such as age, sex, time since onset, having postoperative nasal bleeding or slit punctum, and longer follow-up time did not affect the surgical success in the current patients.

### Several Limitations Should Be Noted in the Current Study

The mean follow-up time in this study was 7.8 ± 1.4 months (a range of 6–13 months). Patients who had no signs of INDO after the 6-month follow-up examination were not asked to return, but advised to visit the clinic if there was a problem. We did find the recurrence of symptoms after an initial success in some patients at 1-year or longer follow-up after surgery. Statistical assessment of these recurrences is, however, beyond the scope of this study. Furthermore, many patients were lost during the long-term follow-up. Future studies with longer follow-ups are still warranted.

The 42 INDO patients who just received topical medication treatment cannot be enrolled into our analysis because their treatment regimen was not uniform and only half of them completed the 6-month follow-up.

The complication of drug residues could not be evaluated fully because the lacrimal outflow system was not syringed routinely until 1 month after surgery. If necessary, we chose to perform lacrimal irrigation at least 2 weeks after the surgery to maximize the drug action.

We did not perform microbial culture for the discharge of our INDO patients because this is not a routine practice in our unit. Microbial analysis may have provided evidence of a nasolacrimal duct infection and helped explain why the ointment is useful for those patients.

The retrospective nature of this study did not allow for data collection that was not mentioned in the medical records, which may lead to a documentation bias, such as the completeness of patients’ history.

## CONCLUSION

Compared with traditional surgical procedures, the PIO surgery is an effective, minimally invasive, timesaving, and easy-to-perform procedure for patients with INDO. Clinicians should be cautious when choosing this surgery for patients with a bilateral onset and discharge. Further prospective, randomized, controlled studies with long follow-ups are still required to demonstrate its efficiency and safety.
